# Lung Ultrasound and Ultrasound Score: A Useful Tool in Neonatal Intensive Care Units for the Diagnosis and Therapeutic Management of Newborns With Respiratory Pathology

**DOI:** 10.7759/cureus.66064

**Published:** 2024-08-03

**Authors:** Alexandra E Popa, Simona D Popescu, Adriana Tecuci, Simona Vladareanu

**Affiliations:** 1 Obstetrics and Gynaecology and Neonatology, Elias Emergency University Hospital, Bucharest, ROU; 2 Obstetrics - Gynaecology and Neonatology, Carol Davila University of Medicine and Pharmacy, Bucharest, ROU

**Keywords:** lung pathology, cardio-thoracic x-ray, surfactant, lung ultrasound (lus), lung ultrasound score

## Abstract

Pulmonary ultrasound has become a fundamental tool for the early detection and management of major neonatal lung diseases in neonatal intensive care units (NICUs). The advantages of this imaging investigation include its rapid execution and information acquisition, non-invasive nature, early diagnosis establishment, dynamic monitoring, and usefulness in therapeutic management. Regarding therapeutic management, the lung ultrasound (LUS) score is used as a basic tool for indicating surfactant administration.

Performing and interpreting pulmonary ultrasounds requires an experienced clinician capable of recognizing anatomical structures, understanding the limitations of the technique, and correlating the obtained data with the patient's clinical picture. A series of diagnostic characteristics of pulmonary ultrasonography for neonatal lung pathologies have been described, making pulmonary ultrasound a useful tool in establishing differential diagnoses.

This study evaluates the effectiveness of ultrasonography in determining the severity of lung pathologies in newborns and its impact on therapeutic decision-making, including surfactant administration and continuous positive airway pressure (CPAP) support.

Newborns admitted to the NICU with various respiratory conditions underwent LUS scoring. The study analyzed the relationship between LUS scoring and the severity of conditions such as pneumonia, respiratory distress syndrome, meconium aspiration syndrome, transient tachypnea of the newborn, and pneumothorax. The correlation between LUS scoring, surfactant administration, and CPAP requirements was also examined.

## Introduction

Neonatal pulmonary ultrasound has become a frequently used diagnostic tool for respiratory distress in newborns in recent years, considering that the imaging is non-invasive, can be performed in real-time, and does not involve radiation. It has high sensitivity and specificity, though it requires experience from the examiner.

In 1995, Lichtenstein first described lung ultrasound (LUS) in adults, which was later adapted and used in pediatrics and neonatology [1]. Over the past decade, several studies have demonstrated a considerable increase in the use of ultrasound as an imaging method in neonatology [2].

In the case of premature newborns, respiratory distress occurs in nearly 5.8% of all live births, being among the most important causes of morbidity and mortality in premature newborns, and accounting for 30%-40% of admissions to neonatal intensive care units (NICUs). Management is based on choosing the optimal time to administer surfactant in a timely manner, together with the continuous use of continuous positive airway pressure (CPAP) devices to maintain positive airway pressure. However, although a number of advantages of early surfactant administration are known, the choice of the ideal timing in the first hours of the newborn's life remains a challenge [3].

Neonatal ultrasound (point-of-care ultrasound (POCUS)) was first described in the 1960s, with its uses increasingly reported in the last decade. Neonatal LUS can be utilized in emergency situations, differentiating neonatal respiratory pathologies and thereby reducing neonatal morbidity [4].

The primary objective in managing neonatal respiratory distress syndrome (RDS) is to enhance the survival of the affected neonates through the use of non-invasive respiratory support, surfactant therapy, mechanical ventilation, and comprehensive care for the premature infant. Presently, the European consensus guidelines on RDS recommend the administration of surfactant in neonates receiving non-invasive ventilation when the fraction of inspired oxygen (FiO_2_) exceeds 0.30 during nasal continuous positive airway pressure (nCPAP) at a minimum of 6 cmH_2_O [5].

Furthermore, extensive evidence supports the early administration of exogenous surfactants as crucial in the treatment of RDS. In the long term, this approach not only improves survival rates but also reduces the need for invasive mechanical ventilation, thereby decreasing the incidence of bronchopulmonary dysplasia and mortality [6].

Our study aims to evaluate and correlate the relationship between the score (each lung is divided into three zones: superior anterior, inferior anterior, and lateral; each zone is individually scored on a scale from 0 to 3 points, with a total possible score ranging from 0 to 18 points) obtained from pulmonary ultrasound performed on newborns admitted to the NICU with respiratory pathologies and various constants, such as surfactant administration, the need for mechanical ventilation, or non-invasive respiratory support for these newborns.

## Materials and methods

The conducted study is a prospective observational study. The prospective nature is defined by the mode of including patients in the study, which involved collecting data about the mother from clinical observation sheets, while information about the newborns was obtained through clinical and paraclinical evaluation in the NICU of Elias University Emergency Hospital in Bucharest.

The study was conducted over two years (2022, 2023), including a total of 82 newborns, with gestational ages between 31 and 41 weeks, admitted to the NICU immediately after birth, presenting respiratory functional syndrome diagnosed after clinical and paraclinical investigations.

The database included the following variables for the mother: antenatal corticosteroid administration, mode of birth, and risk factors. In the case of newborns, we analyzed the following variables: gestational age, sex, birth weight, APGAR score at 1 minute and 5 minutes, coloration, presence of respiratory manifestations within the first 24 hours of life, need for additional oxygen - under a cephalic hood, respiratory support/mechanical ventilation, parenteral nutrition, inotropic support, antibiotic therapy, surfactant administration, pulmonary radiological aspect, pulmonary ultrasound aspect, ultrasound score, and number of hospitalization days. Subsequently, data were analyzed to establish the relationship between the ultrasound score and diagnosis (according to severity), surfactant administration, and mode of respiratory support. The newborns were categorized based on their diagnosed respiratory pathology as follows: RDS, transient neonatal tachypnea, meconium aspiration syndrome (MAS), pneumothorax, and pneumonia.

Exclusion criteria included the presence of congenital heart disease, congenital malformations, chromosomal anomalies, or inborn errors of metabolism in newborns.

Upon admission, according to the internal protocol of the neonatology unit, arterial blood gas analyses and chest X-rays were performed. Subsequently, pulmonary ultrasound was performed, and the pulmonary ultrasound score was calculated within the first 24 hours of life.

Pulmonary ultrasound in newborns was performed using a high-frequency linear transducer - 10 MHz, considering that they have a small chest and thinner chest walls, thus achieving better image quality and allowing visualization of the entire lung surface. The ultrasounds were performed in supine, lateral, or lying positions. Each hemithorax was divided into anterior, lateral, and posterior regions relative to the anterior and posterior axillary lines, with longitudinal and transverse scans conducted in all quadrants. Pulmonary ultrasound was performed and interpreted by a clinician certified in ultrasonography with over 10 years of experience in the field of imaging.

In the context of the pathologies included in the study, the following aspects were monitored using ultrasound:

RDS: White lung appearance, presence of more than three confluent B-lines, thickening of the pleural line, and multiple subpleural consolidations.

Transient tachypnea of the newborn (TTN): Symmetrical bilateral distribution of B-lines with a regular pleural line and the presence of the double lung point.

Pneumothorax: Absence of lung sliding (corresponding to the stratosphere sign or barcode sign in M-mode examination), absence of B-lines with the presence of A-lines, presence of lung point, and absence of lung pulse.

MAS: Pulmonary consolidation with air bronchogram, pleural line abnormalities with disappearance of A-lines, atelectasis, pleural effusion, and interstitial syndrome (B-lines).

Congenital pneumonia: Pleural line abnormalities, subpleural consolidation (hepatization appearance with the presence of air bronchogram), interstitial syndrome (more than 3 B-lines in the intercostal space), and disappearance of lung sliding associated with the presence of lung pulse.

Radiological aspects found in the syndromes included in the study

In TTN, the following radiologic features have been identified: interstitial edema predominantly perihilar, pleural effusions, and perihilar alveolar opacities.

In MAS, chest X-ray findings reveal increased lung volume, irregular asymmetric pulmonary opacities, pleural effusions, pneumothorax or pneumomediastinum, and multifocal consolidation.

In RDS, the characteristic radiologic signs include decreased lung transparency with diffuse atelectasis, classically described as having a reticulogranular or ground-glass appearance, progressive diminution of the cardiac silhouette contour, and the presence of air bronchograms.

In congenital pneumonia, radiological aspects include localized or diffuse alveolar densities, reticular opacities, and features similar to RDS. The most frequent and characteristic alveolar pattern is a dense bilateral air space-filling process with numerous air bronchograms.

In the case of pneumothorax, the hyperlucent hemithorax sign is seen in the anterior pneumothorax, and the medial stripe sign and/or sharp mediastinum sign are observed in the medial pneumothorax.

Interpretation of LUS scoring

Score 0: A-lines, up to two B-lines, and a normal pleural line (indicating normal lung aeration).

Score 1: Three or more non-confluent B-lines (indicating interstitial syndrome).

Score 2: Confluent B-lines with or without subpleural consolidations less than 1 cm and a thickened pleural line (indicating severe interstitial syndrome or a "white lung" appearance).

Score 3: Extensive consolidations greater than 1 cm [7].

Most newborns received surfactant via the INSURE method (intubate-surfactant-extubate), subsequently attempting respiratory support through non-invasive CPAP.

Statistical analysis

Statistical analysis was performed using IBM SPSS Statistics 29.0.1.1 (IBM Corporation, Armonk, NY, 2024). Continuous variables were expressed as mean ± standard deviation. Descriptive statistics were used to describe the sample in terms of the number of patients, as the sample was smaller than 100 patients, and analytical statistics were also employed. The Student's t-test for independent groups was used for data comparison, regardless of their distribution, as the groups were larger than 20 patients. A p-value <0.05 was considered statistically significant. For variables not normally distributed, the Mann-Whitney U test for independent groups with Yates correction was used. To assess the equality of variances of a variable in two groups, Levene's test was employed. If the p-value of Levene's test was <0.05, the value of t for unequal variances was reported. For categorical variables, the Pearson chi-square test was applied to determine if there was a statistically significant difference (p<0.05) between the expected and the observed frequencies in the two groups.

## Results

**Figure 1:**
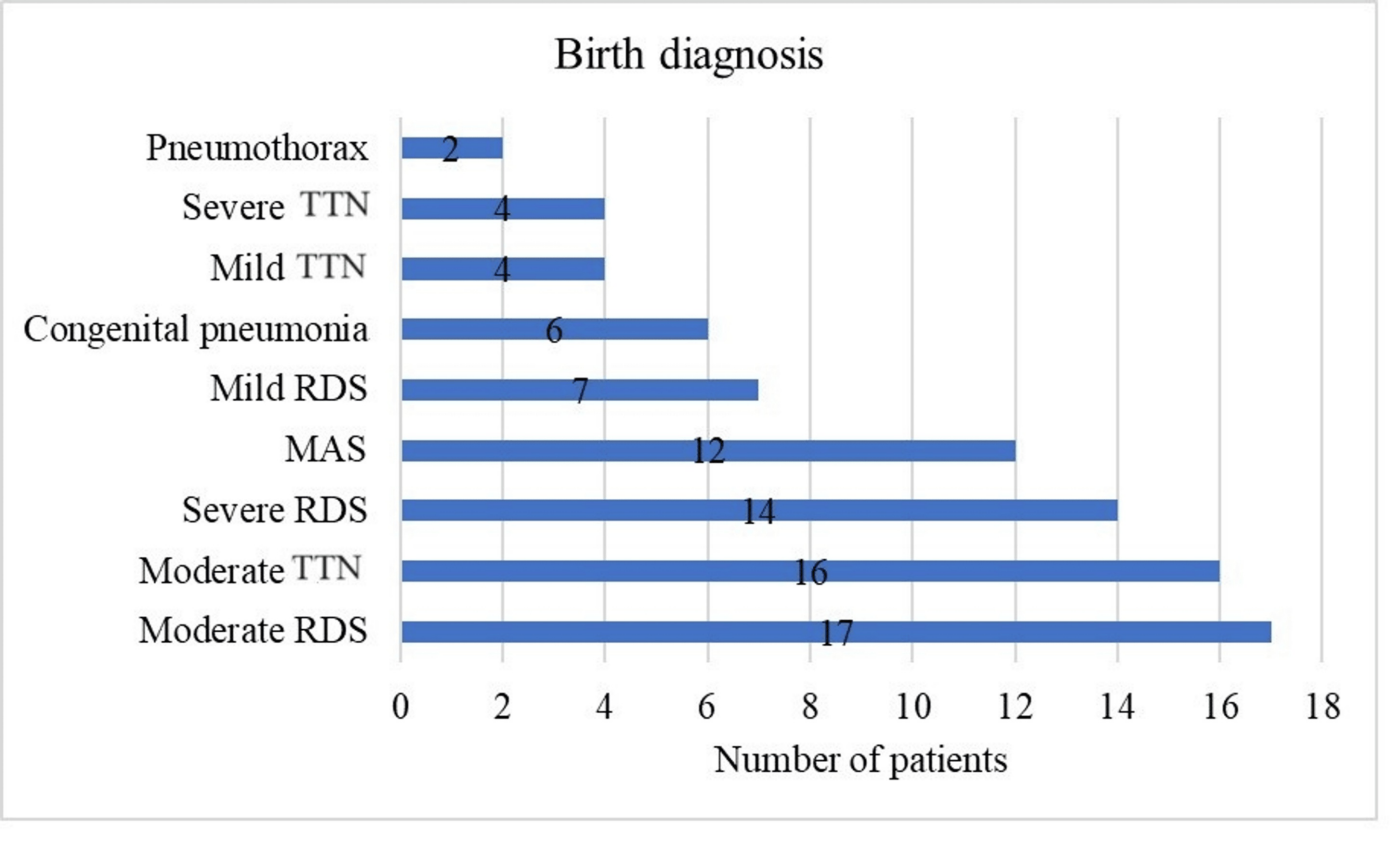
Graphical representation of the diagnosis at birth TTN: transient tachypnea of the newborn; RDS: respiratory distress syndrome; MAS: meconium aspiration syndrome.

Regarding respiratory pathology at birth, out of the total of 82 newborns included in the study, most presented with moderate RDS (n = 17), followed by moderate TTN (n = 16). Fourteen patients had severe RDS. Twelve patients had MAS. Seven patients had mild RDS. Six patients were diagnosed with congenital pneumonia. Four patients each had severe TTN and mild TTN, respectively, and only two patients were diagnosed with pneumothorax (Figure 1).

To objectively assess the severity of pathologies in our study, we used the Silverman-Andersen Respiratory Severity Score. It is a tool designed to evaluate the severity of respiratory distress in neonates. The score quantifies respiratory severity by evaluating five parameters of the work of breathing, and it assigns an overall score ranging from 0 (no respiratory distress) to 10 (severe respiratory distress). Each of the five parameters is scored individually from 0 to 2. The scores for each parameter are then summed to obtain a total score. The total score ranges from 0 to 10, with higher scores indicating greater severity of respiratory distress. Interpretation of scores is as follows: 0: no respiratory distress; 1-3: mild respiratory distress; 4-6: moderate respiratory distress; 7-10: severe respiratory distress [8].

**Figure 2:**
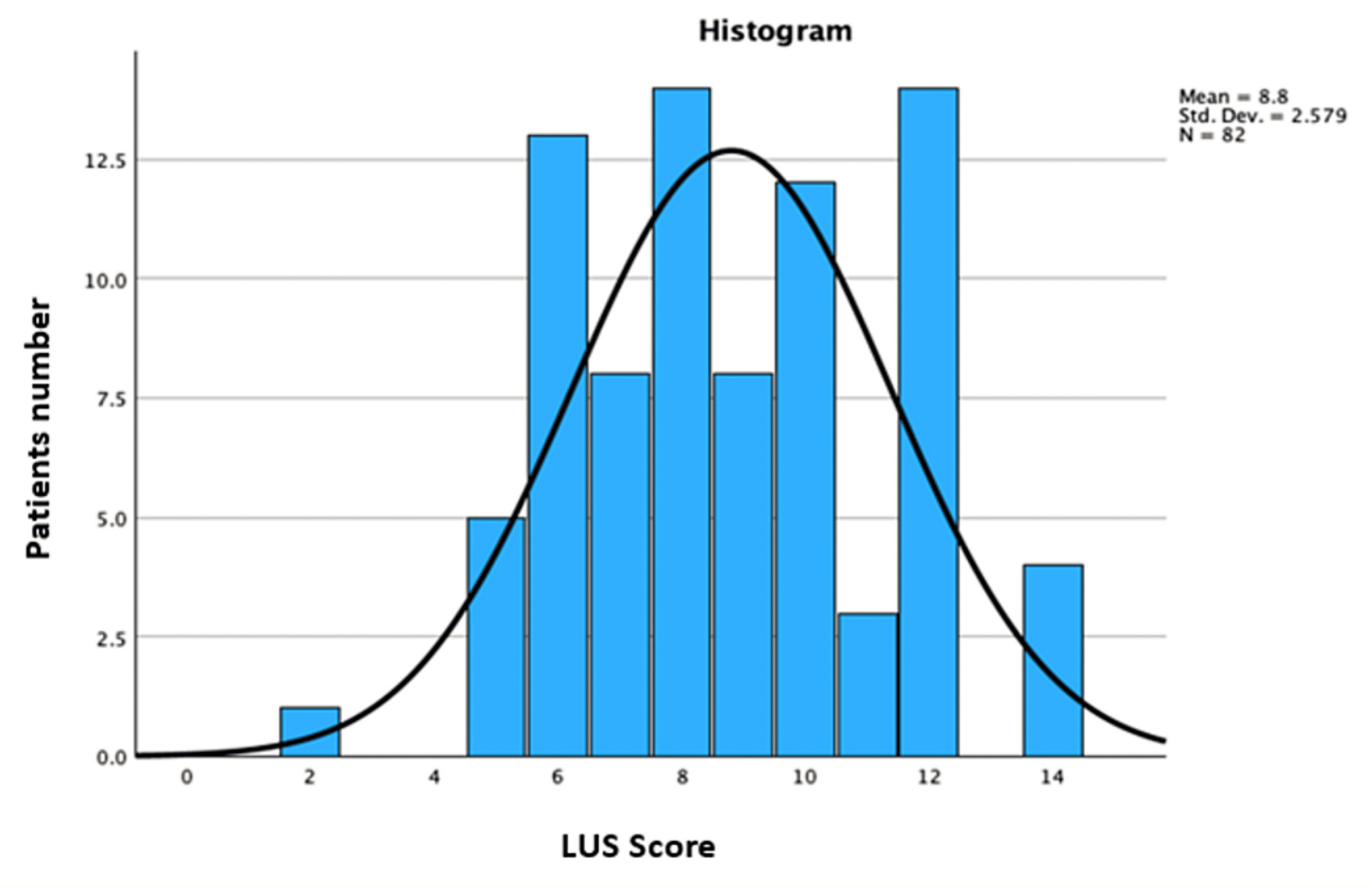
Distribution and average LUS score in the sample LUS: lung ultrasound.

The average LUS score in the cohort was 8.80 ± 2.57, with a minimum score of 2 and a maximum score of 14 (Figure 2).

**Figure 3:**
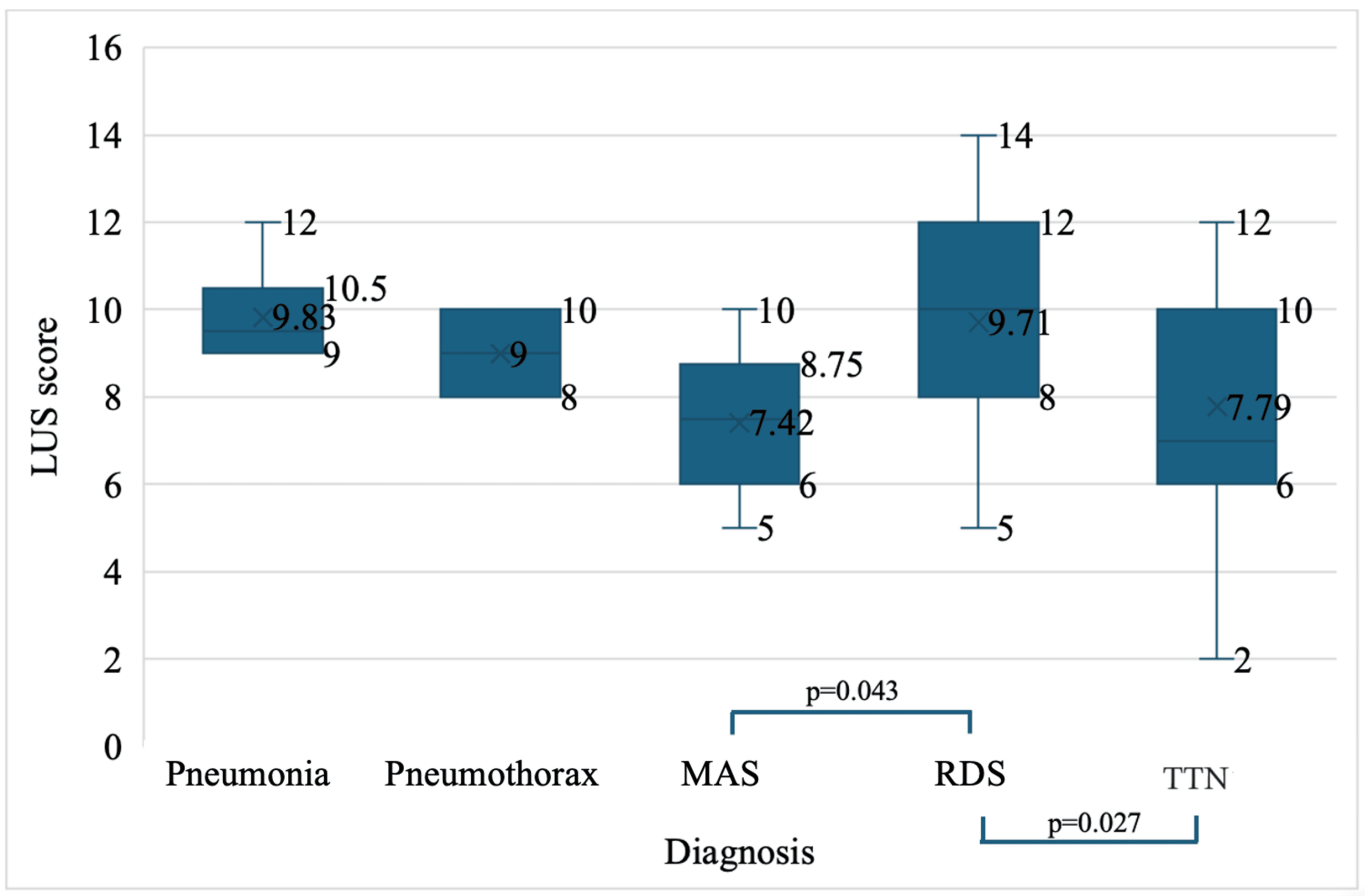
Average LUS score by pathology LUS: lung ultrasound; MAS: meconium aspiration syndrome; RDS: respiratory distress syndrome; TTN: transient tachypnea of the newborn.

The average LUS scores, in descending order, were as follows: pneumonia (9.83 ± 1.16), RDS (9.71 ± 2.68), pneumothorax (9.00 ± 1.41), TTN (7.79 ± 2.51), and MAS (7.42 ± 1.73). A one-way analysis of variance (ANOVA) test was conducted to determine if the LUS score differed by diagnosed disease. The cohort was diagnosed with five diseases, and the mean scores according to diagnosis are presented in Figure 3. The ANOVA test showed statistically significant differences between the types of diagnosed diseases in the group (F(4, 77) = 3.62, p = 0.009) (Figure 3).

Additionally, significant differences were observed between groups, specifically between patients with TTN and patients with RDS (p = 0.027), and between patients with RDS and patients with MAS (p = 0.043).

**Figure 4:**
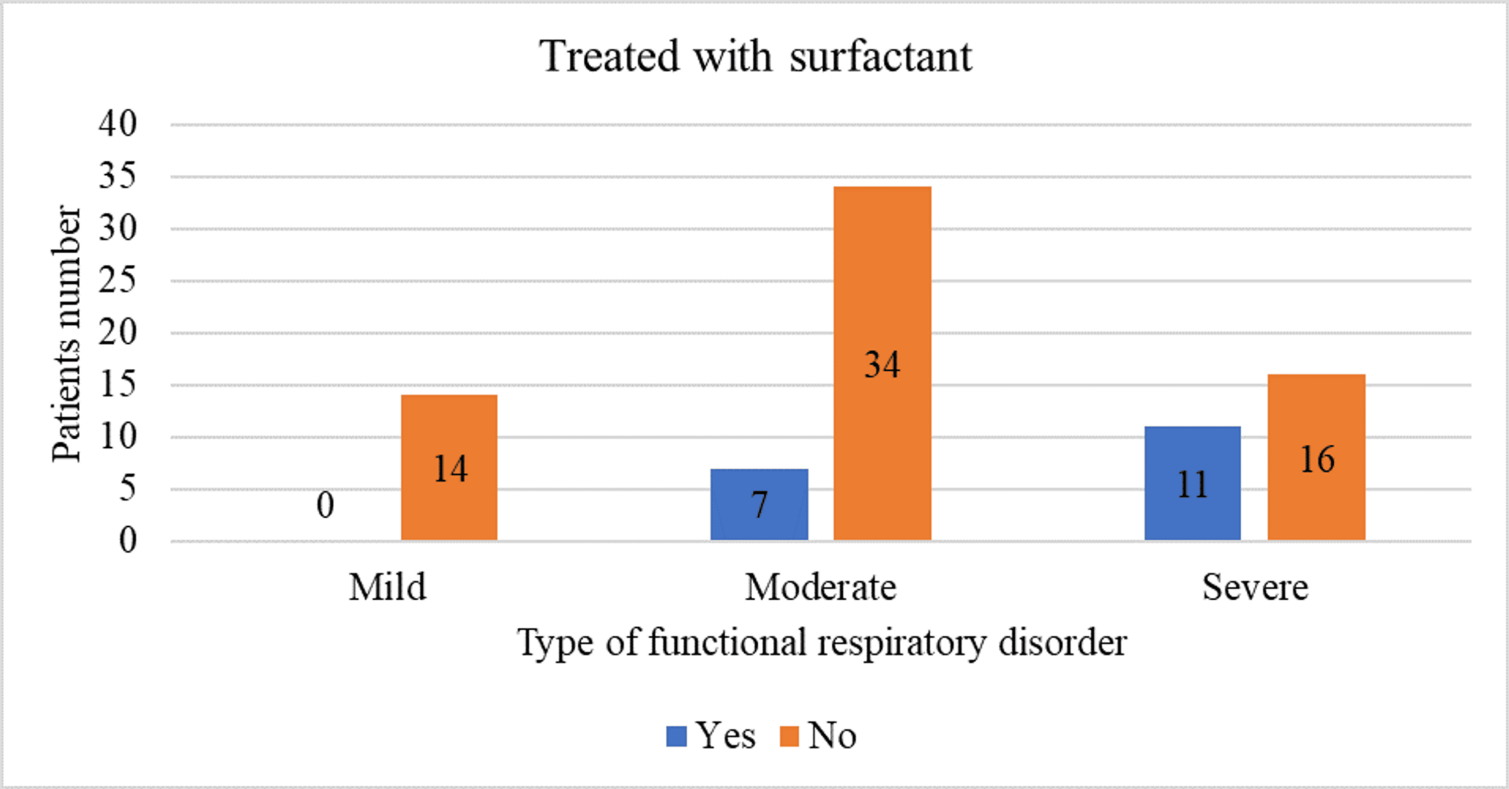
Sample distribution by type of respiratory syndrome and surfactant administration

Significant differences were found in patients who received surfactant treatment (χ²(2) = 10.071, p = 0.007). Specifically, no patients with mild respiratory functional syndrome received surfactant, seven children with moderate syndrome received it, and 11 with severe syndrome received it. These data are graphically illustrated in Figure 4.

Further analysis was conducted to determine if there were statistically significant differences between two groups of patients - those who received surfactant (n = 18) and those who did not (n = 64) - regarding gestational age, birth weight, Apgar score at 1 minute, and Apgar score at 5 minutes. The first step was to determine if these numerical variables were normally distributed in the two patient groups using the Shapiro-Wilk test. As seen in Table 1, only birth weight was normally distributed in both patient groups (p > 0.05). Therefore, the non-parametric Mann-Whitney test was conducted to analyze the existence of statistically significant differences between the two groups concerning gestational age, Apgar score at 1 minute, and Apgar score at 5 minutes. To compare if there were significant differences in birth weight, the independent t-test was used (Table 1).

**Table 1:** Shapiro-Wilk test results by surfactant administration df, degrees of freedom.

Variable	Surfactant administration	Statistic	df	p-Value
Gestational age	Yes	0.862	18	0.013
	No	0.937	64	0.003
Birth weight	Yes	0.970	18	0.803
	No	0.970	64	0.124
Apgar score at 1 minute	Yes	0.613	18	<0.001
	No	0.723	64	<0.001
Apgar score at 5 minutes	Yes	0.775	18	<0.001
	No	0.772	64	<0.001

Gestational age was significantly different between the two groups of patients (those who received surfactant, n = 18, and those who did not receive surfactant, n = 64) with U =348.00, Z =-2.575, and p =0.010. Similarly, the Apgar score at 1 minute was significantly different (U = 352.00, Z = -2.866, p = 0.004), as was the Apgar score at 5 minutes (U = 334.00, Z = -3.056, p = 0.002).

A visual inspection of Figures 2-4 suggests a different distribution of the aforementioned numerical variables in the two groups. Therefore, the statistical analysis aimed to examine differences based on distribution rather than mean.

**Figure 5:**
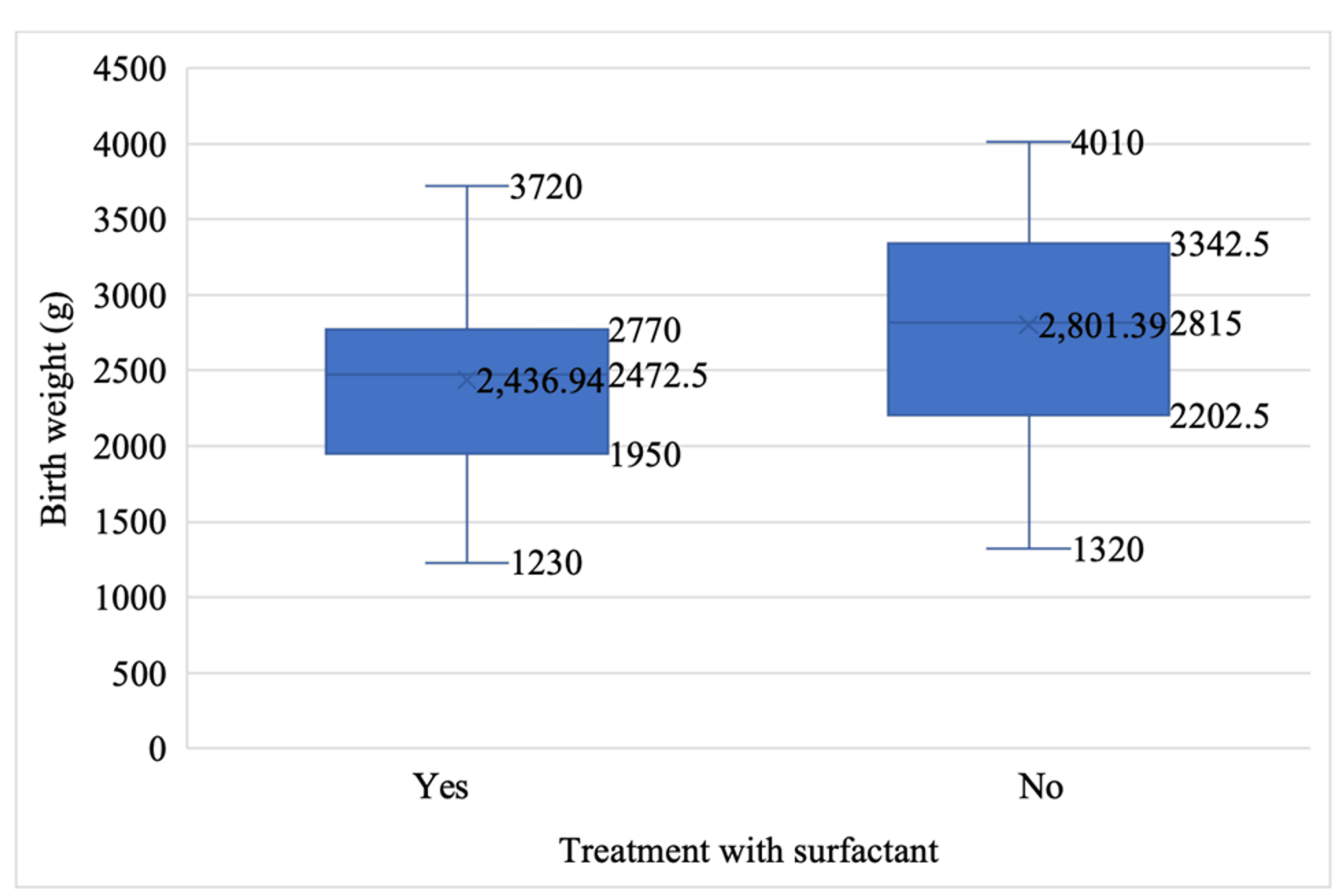
Average birth weight based on surfactant administration

The mean birth weight in the group of children who received surfactant was 2436.94 ± 669.83 g, which was lower than that of the group of children who did not receive surfactant, whose mean birth weight was 2801.39 ± 723.37 g (Figure 5). However, this difference was not statistically significant (t(80) = -1.918, p = 0.059).

**Figure 6:**
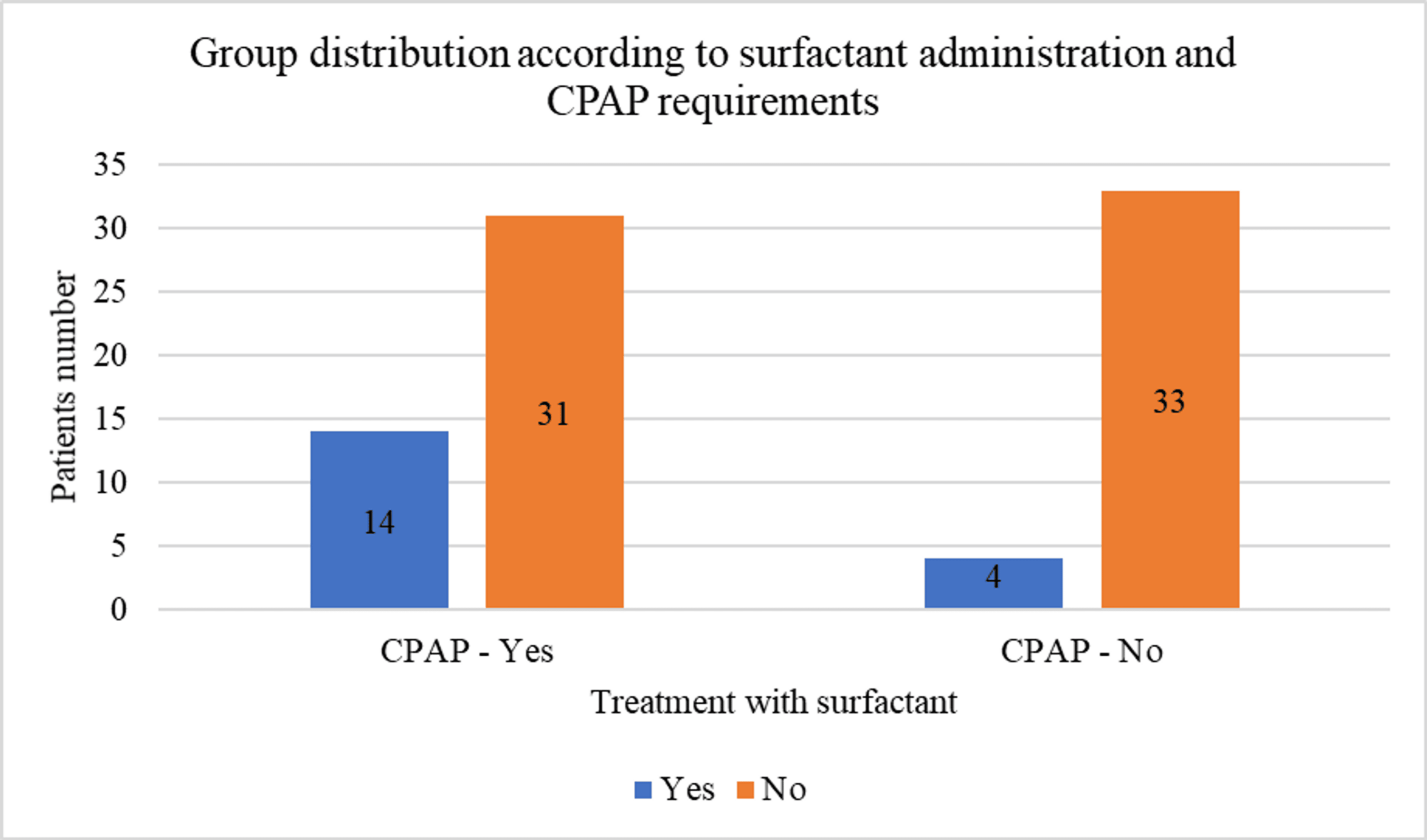
Sample distribution by CPAP requirement and surfactant administration CPAP: continuous positive airway pressure.

Additionally, the majority of newborns who required respiratory support via CPAP did not receive surfactant (n = 31). These data are comparable to the patients who required CPAP therapy and also received surfactant (n = 14). Thus, the differences were statistically significant (χ²(1) = 4.884, p = 0.027), and the data are illustrated in Figure 6.

**Figure 7:**
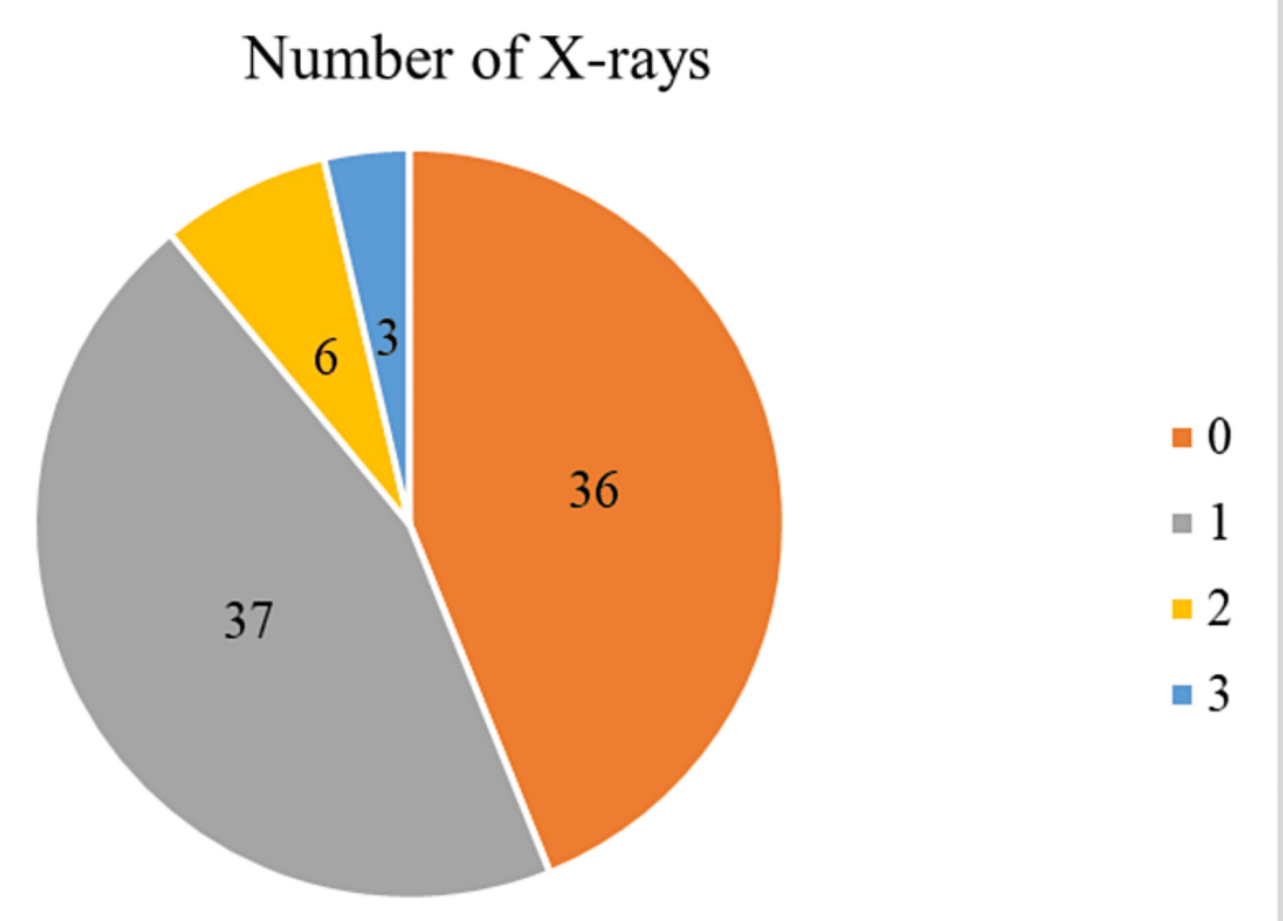
Sample distribution by the number of chest X-rays performed

In Figure 7, the number of chest X-rays performed on the patients is represented, showing the following aspects: 36 newborns did not undergo any chest X-rays, 37 had one chest X-ray during their hospitalization, six newborns had two chest X-rays, and three had three chest X-rays during their hospitalization.

**Figure 8:**
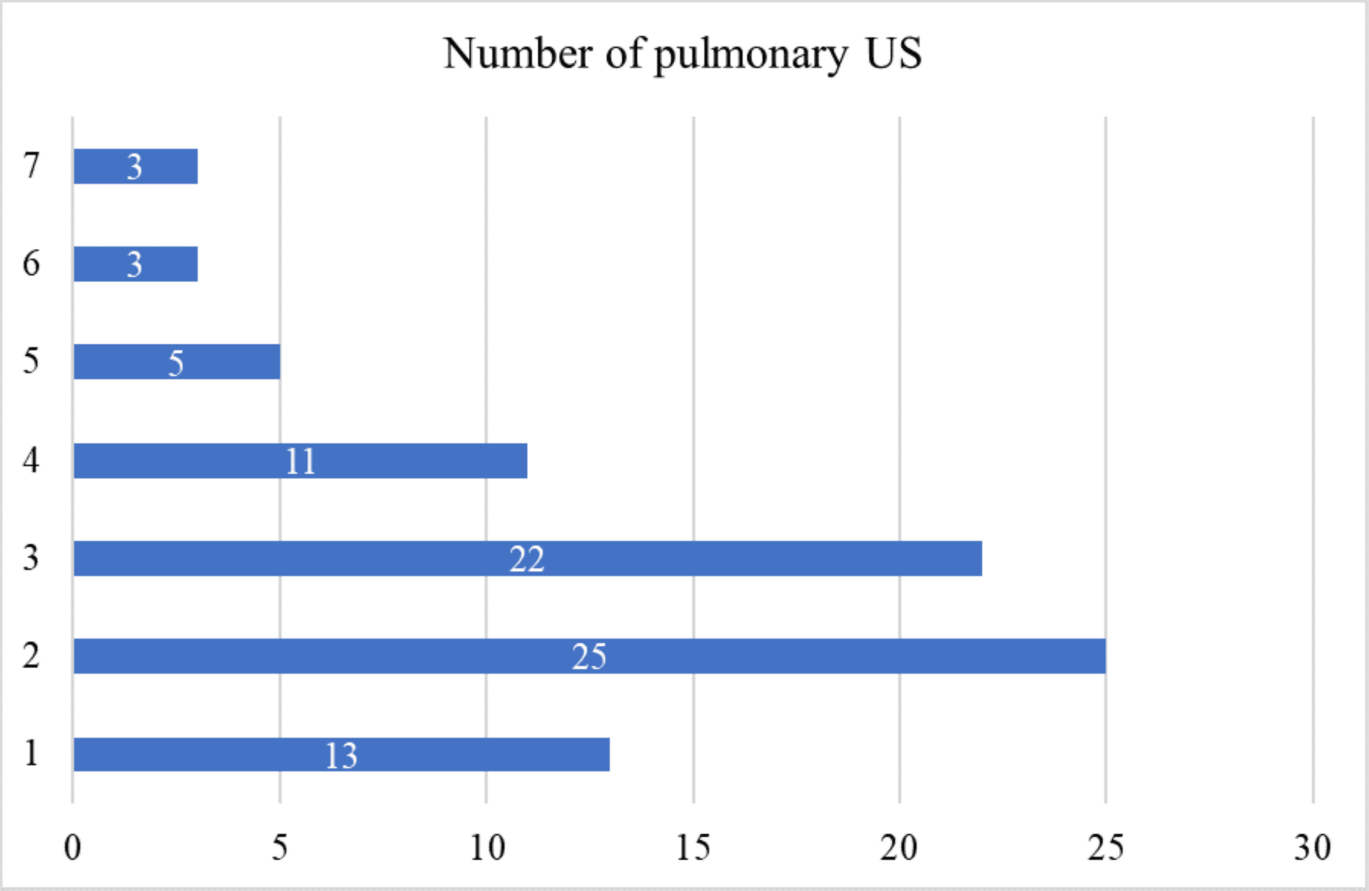
Sample distribution by the number of lung ultrasounds performed

Regarding pulmonary ultrasound, most newborns underwent two pulmonary ultrasounds (n = 25), while 22 underwent three pulmonary ultrasounds. Out of the total newborns, only three required six or seven pulmonary ultrasounds (Figure 8).

**Figure 9:**
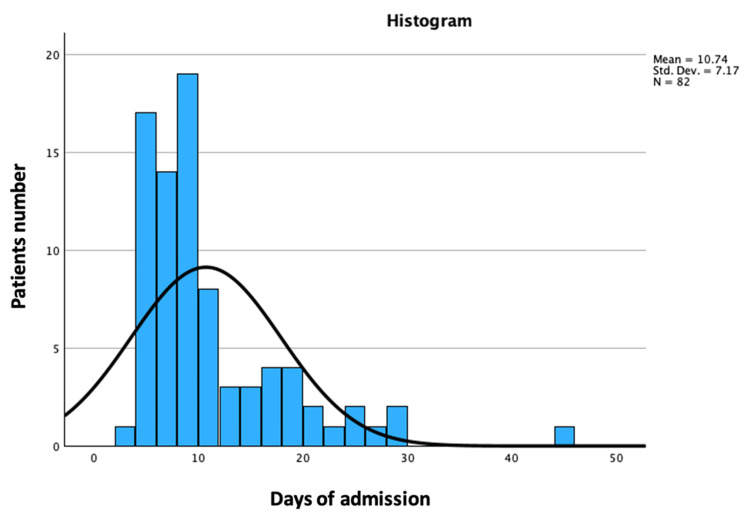
Average number of hospitalization days in the sample

The average number of days spent in the hospital by each newborn was 10.74 ± 7.17 days (Figure 9).

## Discussion

Observing the trend in diagnosing major neonatal lung pathologies through lung ultrasound has revealed an increase in the use of lung ultrasound for diagnostic purposes and its dynamic monitoring.

In this article, we present the statistical data obtained from the Elias University Emergency Hospital Clinic in Bucharest, conducted over four years, regarding the use of lung ultrasound as a first-line imaging method in the NICU. We compare this data with that obtained through the most commonly used imaging method today, which is cardiopulmonary radiography. We discuss the advantages and disadvantages of both methods, the associated risks, and their impact on the newborn and their pathology.

Currently, although there is no protocol regarding the first-line use of lung ultrasound over radiography, developed countries are increasingly studying this possibility. By combining the current progress of research and the clinical experience from our study, we can outline some aspects regarding the routine use of lung ultrasound, especially in NICUs.

Respiratory pathologies in newborns represent the most frequent indication for admission to the NICU and are also the most common cause of early morbidity and mortality in newborns [9,10].

LUS score by diagnosis

The analysis of LUS scores revealed critical insights into the severity of various neonatal respiratory conditions. Higher LUS scores were associated with more severe lung pathology. For instance, pneumonia and RDS exhibited the highest LUS scores, indicating significant lung involvement. This is supported by the statistically significant differences observed between conditions such as MAS and RDS, as well as TTN and RDS. These findings underscore the value of LUS as a diagnostic tool in assessing the severity of neonatal respiratory conditions and guiding therapeutic decisions like surfactant administration.

Surfactant treatment and CPAP requirements

The data highlighted that the administration of surfactant is more common in severe cases, which often also require CPAP support. Mild cases generally did not receive surfactant, aligning with clinical guidelines that reserve this treatment for significant respiratory distress. This trend emphasizes the importance of evaluating the severity of the respiratory disorder to determine the need for surfactant treatment.

Statistical analysis of variables related to surfactant administration

Significant differences were observed in gestational age and Apgar scores between neonates who received surfactant and those who did not, indicating that these factors are crucial in the decision-making process for surfactant administration. Conversely, birth weight did not show a significant difference, suggesting that it is less critical in this context.

Birth weight distribution in relation to surfactant administration

Newborns treated with surfactant generally had lower birth weights, pointing to a higher likelihood of requiring surfactant treatment among lighter and potentially more premature infants due to their increased vulnerability to respiratory complications.

Number of X-rays performed

The majority of neonatal patients underwent either no X-rays or a single X-ray during their hospitalization, reflecting a preference to minimize radiation exposure. Multiple X-rays were reserved for more severe or complicated cases, necessitating detailed and frequent monitoring.

Number of pulmonary ultrasounds

Most patients had two or three pulmonary ultrasounds, indicating regular monitoring and adjustment of treatment plans based on ultrasound findings. Fewer patients required four to seven ultrasounds, pointing to more severe or complex cases needing extensive follow-up.

Length of hospital stay

The average length of hospital stay was around 10 days, with most patients having shorter stays and a minority requiring extended hospitalization due to more severe conditions. This distribution highlights the efficiency of clinical interventions and the effectiveness of LUS in guiding timely treatment.

Conclusions

The conclusion of our study is that the findings emphasize the critical role of LUS in diagnosing and managing neonatal respiratory conditions. Higher LUS scores correlate with more severe pathology, guiding therapeutic decisions such as surfactant administration. The study underscores the importance of minimizing radiation exposure through limited X-rays and highlights the significance of birth weight and gestational age in determining surfactant treatment needs. Regular monitoring using pulmonary ultrasound ensures effective neonatal care, allowing for timely and appropriate interventions based on the severity and progression of each case.

In 2015, Brat and colleagues published the first study aiming to demonstrate the predictive capacity of neonatal lung ultrasound for the need for exogenous surfactant administration in newborns with RDS. The study included 130 newborns and, through the LUS score performed after admission to neonatal intensive care and before surfactant administration, they objectified that there were significant correlations between the obtained score and the oxygenation level of the newborns, as well as a good reliability of the ultrasound in predicting surfactant administration in premature newborns with a gestational age of <34 weeks on nCPAP from birth. Thus, surfactant could be administered in a timely manner before the worsening of these newborns' symptoms [11].

A similar prospective study was published by De Martino et al. regarding the predictive accuracy of lung ultrasound for the need for exogenous surfactant therapy in premature newborns with a gestational age of ≤30 weeks. Anterior and lateral chest wall scans were performed. The result of this study suggested that the LUS score is significantly correlated with the oxygenation index [12].

In our study, we analyzed the fraction of inspired oxygen (FiO_2_) for each case. Our findings were consistent with the literature. Thus, when FiO_2_ exceeded 0.30 (30%) during the use of nCPAP at a minimum of 6 cmH2O, it indicated significant respiratory distress and was associated with the need for surfactant administration in most cases.

Perri et al. enrolled 56 newborns with a gestational age of approximately 31 weeks in a prospective study and compared the scores obtained from lung ultrasound and cardiothoracic radiography to predict early surfactant administration in newborns with RDS. Ultrasound scores were better than X-ray scores in the early recognition of newborns with RDS who require surfactant administration. Subsequently, they dynamically evaluated the patients 2 h after surfactant administration and discovered the utility of ultrasound in predicting the need for a repeat dose of exogenous surfactant. An LUS score ≥7 showed a sensitivity of 94% and a specificity of 60% [13].

The same hypothesis was studied in a double-blind prospective study by Vardar et al., conducted on newborns with RDS with a gestational age of <34 weeks who presented clinical and radiological signs, and they found a significant correlation between ultrasound results and the need for surfactant administration as well as the number of doses. The LUS threshold score that predicted the need for surfactant administration was 4, with a sensitivity of 96% and a specificity of 100% [14].

A meta-analysis that included 486 newborns also confirmed the accuracy of LUS in guiding surfactant replacement, and infants with LUS score >5-6 had a significantly increased risk of surfactant treatment compared to infants with LUS <5-6. LUS, particularly the LUS score, can be used accurately to determine the need for surfactant replacement therapy or mechanical ventilation in infants with respiratory distress treated with NCPAP support. The accuracy is better in smaller premature infants compared to late preterm and term infants [15].

To evaluate the diagnostic capability of LUS in detecting the pulmonary manifestations of neonatal RDS, as well as to monitor treatment response, 100 newborns with clinical and radiographic signs of RDS were included in this prospective study. LUS was performed using both a transthoracic and a transabdominal approach within the first 24 hours of life and subsequently to detect pulmonary manifestations and monitor treatment response. LUS findings were compared with chest X-ray results. Compared to chest X-ray, LUS had a sensitivity of 98% and a specificity of 92% in detecting pulmonary manifestations of RDS. Following treatment response, LUS had a sensitivity of 100% and a specificity of 94%. LUS can be an alternative imaging diagnostic method to chest X-ray in follow-up newborns with RDS and can reduce subsequent radiation doses [16].

Ultrasonographic and radiological aspects in the most frequent neonatal pulmonary pathology

High specificity and sensitivity are noted when using lung ultrasound versus chest X-ray in diagnosing and monitoring progress in neonatal pneumothorax or pleural effusions [17]. Thus, the specificity and sensitivity of lung ultrasound in the diagnosis of neonatal pneumothorax were 98% and 99%, respectively. However, chest X-ray has always been used as a reference standard, with a sensitivity of 82% and a specificity of 96%. Studies have shown that the sensitivity of lung ultrasound in diagnosing pneumothorax, as a sole diagnostic criterion, except for chest X-ray, was 98% and the specificity was 100%, demonstrating greater accuracy than chest X-ray. In conclusion, lung ultrasound had better sensitivity and specificity than chest X-ray in diagnosing pneumothorax [18].

Other studies of this pathology have found that lung ultrasound is at least as useful as chest X-ray in diagnosing neonatal pneumothorax, but when comparing the two diagnostic methods, ultrasound presented better sensitivity and specificity in the early detection of neonatal pneumothorax. However, further prospective studies are needed to substantiate the practical value of ultrasound in diagnosing neonatal pneumothorax [19-21].

In a 2021 meta-analysis conducted by He L, Sun Y, Sheng W, and Yao Q, which included approximately 1514 newborns, the authors concluded that LUS has high specificity and sensitivity for diagnosing TTN [22]. Another multicentric study by F. Raimondi and colleagues evaluated 65 newborns with a gestational age between 34 and 40 weeks who clinically presented with neonatal tachypnea. Lung ultrasounds were performed within the first 60-180 minutes of life and subsequently at intervals of 6-12 h if symptoms persisted. The authors found that 47.6% of these patients had the double lung point sign, a regular pleural line without present consolidations [23].

In a study conducted in Cairo during 2011-2012 on 75 newborns admitted to intensive care with clinical signs of pneumonia, chest X-ray results confirmed the diagnosis in 64 cases (85.3%), while ultrasound confirmation was in 68 patients (90.6%). Bedside lung ultrasound proved extremely sensitive, specific, and reproducible for excluding the underlying pneumonic process, as well as for early detection and monitoring of possible complications, potentially serving as an alternative to chest X-ray without exposing these newborns to radiation [24]. A sensitivity of 100% and a specificity of 100% were achieved by demonstrating the presence of large regions of lung consolidations with irregular margins and air bronchograms as one of the diagnostic criteria for neonatal pneumonia [25]. The LUS score for newborns with infectious pneumonia can be applied to assess disease severity [26]. It can also be considered an alternative for diagnosing ventilator-associated neonatal pneumonia [27].

Lung ultrasound in the case of MAS, using chest X-ray as a reference, Piastra M and colleagues examined six newborns with MAS in the first hours of life. They found confluent or sparse B-lines, lung consolidation, atelectasis, and the presence of air bronchograms, which corresponded to radiological findings. In conclusion, they found that ultrasound images are promising for diagnosing and monitoring patients with MAS [28]. In another relatively large-scale study involving 117 newborns with MAS and 100 control newborns, similar radiological and ultrasound characteristics were detected, and irregular subpleural consolidations with air bronchograms showed 100% sensitivity and specificity in diagnosing MAS [29]. Irregular lung consolidations detected by ultrasound can also be found in pneumonia cases but are usually bilateral in MAS and unilateral in pneumonia, making it relatively easy to differentiate between the two pathologies [30].

## Conclusions

According to the data obtained in our study, based on the LUS score, we can establish the diagnosis, the severity of the disease, and the necessity for surfactant administration. Together with the other analyzed parameters, we can conclude that the LUS score is an important predictive factor that can guide the management of cases in NICUs.

Neonatal LUS has several advantages: it is non-invasive, radiation-free, and can be performed quickly and portably at the patient's bedside. Due to these benefits, special programs have been implemented in NICUs to encourage its use. Consequently, the number of X-rays, previously regarded as the gold standard, has been reduced by half, decreasing the radiation exposure for newborns.
